# The non-linear relationship between serum albumin and diabetic retinopathy in type 2 diabetes mellitus: a secondary analysis based on a cross-sectional study

**DOI:** 10.1186/s12886-024-03348-2

**Published:** 2024-03-01

**Authors:** Guo-Qiang Zeng, Yu-Feng Yao, Jian-Bo Zhong, Yi Zhang, Bai-Kang Ye, Xiao-Yan Dou, Li Cai

**Affiliations:** 1 Department of Ophthalmology, Shenzhen University General Hospital, Xueyuan AVE 1098, Nanshan District, Shenzhen, Guangdong Province China; 2 Shenzhen University Health Science Center, 518000 Shenzhen, Guangdong Province China; 3 Department of Ophthalmology, Shenzhen Second People’s Hospital, The First Affiliated Hospital of Shenzhen University, No.3002 Sungang West Road, 518035 Shenzhen, Guangdong Province China; 4 Shantou University Medical College, No.22 Xinling Road, 515031 Shantou, Guangdong Province China

**Keywords:** Serum albumin, Diabetic retinopathy, Type 2 diabetes, Non-linear, Association

## Abstract

**Background:**

Most studies had shown a linear relationship between serum albumin (sALB) and the prevalence of diabetic retinopathy (DR). Thus, the purpose of this study is to investigate whether their relationship is non-linear.

**Methods:**

We included 426 patients with type 2 diabetes who were hospitalized in Guangdong Provincial People’s Hospital from December 2017 to November 2018. The outcome was the prevalence of DR. A two-piecewise logistics regression model was performed to identify the non-linear relationship between sALB and the prevalence of DR. The inflection point was calculated to determine the saturation effect through the maximum likelihood ratio and a recursive algorithm.

**Results:**

DR was diagnosed in 167 of 426 type 2 diabetic patients. The relationship between sALB and DR was nonlinear. When sALB was less than 38.10 g/L, a significant negative association was observed (OR = 0.82; 95% CI, 0.72–0.94; *P* = 0.0037), while no significant association was observed when sALB was greater than 38.10 g/L (OR = 1.12; 95% CI, 0.92–1.35; *P* = 0.2637).

**Conclusions:**

The relationship between sALB and the prevalence of DR is non-linear. sALB is negatively associated with the prevalence of DR when sALB is less than 38.10 g/L. Our findings need to be confirmed by further prospective research.

## Introduction

Diabetic retinopathy (DR) is a highly specific neurovascular complication of diabetes [1]. Its prevalence is closely related to the levels of glycemic and the duration of diabetes mellitus. In developed countries, DR is the leading cause of blindness in the population aged 20–74 years. According to the latest analysis, the global prevalence of DR will reach 160.5 million by 2045 [2]. The pathogenesis of DR is a highly complex process involving hyperglycemia, leukocyte arrest, ischemia, and inflammation [3]. Traditional risk factors for the development of DR include long diabetes duration, hypertension, insulin use, high HbA1c levels, triglycerides, and low-density lipoprotein cholesterol [4, 5].

A recent study found a significant negative correlation between serum albumin (sALB) levels and the prevalence of DR, suggesting that sALB is negatively correlated with the prevalence of DR [6], which draws our attention to sALB as a bio-marker of DR. sALB is synthesized by hepatocytes and it is the most abundant protein in plasma. It can bind and transport endogenous or exogenous substances to maintain the plasma colloid osmotic pressure. Moreover, sALB plays a key role in clearing free radicals, inhibiting platelet aggregation, and anticoagulation [7, 8]. It is well understood that oxidative stress and inflammatory response are key factors in the development of DR in patients with type 2 diabetes mellitus [9–11]. Both the increased oxidative stress and inflammatory response caused by low sALB levels may be the primary mechanism of DR progression [12, 13].

Most studies drew their conclusion about the relationship between sALB and the prevalence of DR based on the linear analysis [6, 14]. However, the relationship between exposure factors and outcomes tends to be nonlinear in clinical research. In this event, we need more effective methods to deal with the nonlinear association.

In this study, we performed a secondary data analysis based on existing data from a published paper [15]. sALB was used as an independent variable, and the outcome variable was the prevalence of DR. We aimed to investigate the relationship between sALB and the prevalence of DR and validate whether it is nonlinear.

## Method

### Data source and study population

The data came from the “Dryad” database (https://datadryad.org). This website allows users to free download raw data from the literature. We cite the corresponding Dryad data package in this paper following the Dryad terms of service. [Zhuang, X et al. (2019). Data from Association of diabetic retinopathy and diabetic macular edema with renal function in southern Chinese patients with type 2 diabetes mellitus: a single-center observational study, Dryad, Dataset, 10.5061/dryad.6kg1sd7]. The study was a retrospective cross-sectional study that included 426 patients with type 2 diabetes who visited Guangdong Provincial People’s Hospital’s endocrine department and received an ophthalmology consultation from December 2017 to November 2018. The exclusion criteria for the study population were: (1) other eye diseases affecting ocular circulation such as glaucoma, endophthalmitis, retinal vascular obstruction, age-related macular degeneration, refractive error > 3.00D, and ocular trauma; (2) the previous history of intravitreal drug injections or renal dialysis; (3) severe systemic diseases such as myocardial infarction, cerebral infarction, and connective tissue disease; (4) women in pregnancy or menstrual status. More details of the study can be found in the study completed by Zhuang X et al [15]. Zhuang X et al. clearly stated that: this study was performed according to the Declaration of Helsinki and approved by the Research Ethics Committee of Guangdong Provincial People’s Hospital (registration number: gdrec2016232A).

### Variable source and definition of DR

All clinical information is obtained through the electronic medical record. Laboratory tests include liver and kidney function, lipid analysis, and urinalysis. Blood and urine samples were collected when patients were fasting before 8:00 p.m. DR was diagnosed by a fundus specialist using fundus photography. DR was classified into five groups based on the international clinical diabetic retinopathy grading criteria [16]: (1) no retinopathy; (2) mild non-proliferative diabetic retinopathy (NPDR); (3) moderate NPDR; (4) severe NPDR; and (5) proliferative diabetic retinopathy (PDR). 2)-5) were defined as the presence of DR in this study. Zhuang X et al [15] described the measurement assessment methods and criteria for each variable in detail in the original.

### Statistical analysis

The clinical characteristics of participants were described and divided into two groups based on whether they had DR or not. For continuous variables with normal distribution, data were presented as “mean standard deviation” with p-values obtained by t-test for two independent samples. For continuous variables with non-normal distribution, data were presented as “median (Q1-Q3)” with p-values obtained by the Kruskal Wallis rank sum test. And for categorical variables, data were presented as “sample size (percentage)” with p-values obtained by χ^2^ test. The logistic regression model was used to analyze the relationship between sALB and the prevalence of DR. First, sALB was analyzed as a continuous variable, and then sALB was divided into four groups based on quartiles to further validate the relationship. The unadjusted, minimally adjusted, and fully adjusted models were presented following the recommendations of the STROBE statement. Covariates are adjusted if they meet the criteria listed below; (1) Covariate when included or excluded from the model, the odd ratio changes by at least 10% [17]; (2) Covariate was associated with both sALB and DR in clinical practice; (3) Covariate was adjusted in previous similar studies [6]. In addition, the generalized additive model was used to identify the nonlinear relationship between sALB and the prevalence of DR. A two-piecewise logistics regression model was used if a nonlinear correlation was observed between them. To determine whether there was a threshold or saturation effect, the inflection point was calculated through a maximum likelihood ratio and a recursive algorithm based on a two-piecewise regression model. P value less than 0.05 (two-sided) was considered statistically significant in all analyses. The software used for statistical analysis of the data was EmpowerStats version 4.1 (http://www.empowerstats.net, X&Y solutions, Inc. Boston, Massachusetts) and the R language package version 4.2.0 (The R Foundation; http://www.r-project.org; version 4.2.0).

## Results

### The characteristic of participants

The baseline characteristics of the study population are shown in Table 1. A total of 426 patients (240 males and 186 females) participated in the study, with a mean age of 59.00 ± 13.42 years and a median diabetes duration of 10 years (range: 1 to 31 years). 167 patients had DR, with a prevalence of 39.20%. Mild, moderate, and severe NPDR and PDR accounted for 8.92% (*n* = 38), 15.96% (*n* = 68), 7.75% (*n* = 33), and 6.57% (*n* = 28), respectively. Participants with DR had a lower proportion of males, a longer duration of diabetes, a lower BMI, lower levels of sALB, ALT, and AST, higher levels of BUN and D-dimer, and higher rates of hypertension, dyslipidemia, renal insufficiency, and DME than participants without comorbid DR (Table 1).

### The association between sALB and the prevalence of DR

Logistic regression models were used to examine the relationship between sALB and the prevalence of DR. sALB was found to be negatively associated with the prevalence of DR in the crude model (OR = 0.89, 95% CI: 0.84–0.95, *P* = 0.0008). In the minimally adjusted model, the OR was 0.88 (95% CI: 0.82 to 0.95, *P* = 0.0007). In the fully adjusted model, the prevalence of DR was decreased by 8% for each additional 1 g/L of sALB (OR = 0.89, 95% CI: 0.85 to 1.00, *P* = 0.0474, Table 2). We handled sALB as a categorical variable (quartiles) for sensitivity analysis and discovered that the trend of increase was not significant in the fully adjusted model (*P* = 0.0742, Table 2), which suggests the relationship between sALB and the prevalence of DR may be nonlinear.

### The analysis of non-linear association

**Fig. 1:**
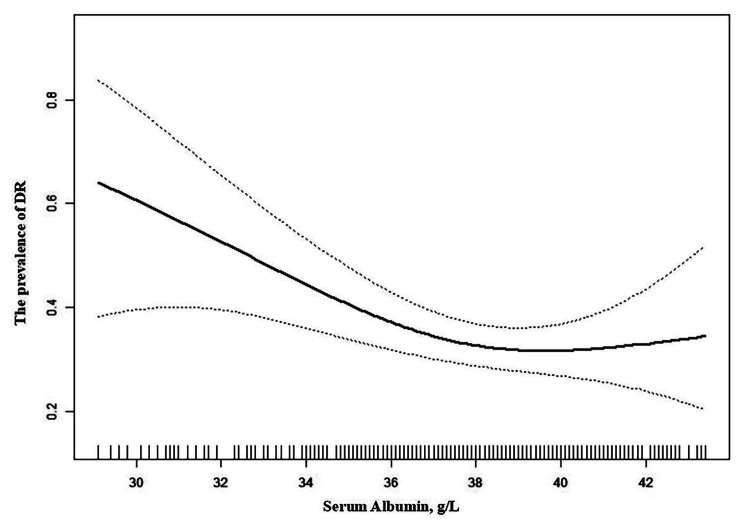
The association between sALB and the prevalence of DR. A non-linear association between them was detected after adjusting for age, sex, DM duration, D-dimer, LDL, serum creatinine, HbA1c, BUN, HBP, and CKD Stage

Figure 1 shows the correlation between ALB and the prevalence of DR is nonlinear after fully adjusted. We calculated the inflection point to be 38.10 g/L through the fully adjusted two-piecewise logistics regression model. ALB was negatively correlated with the prevalence of DR when it was less than 38.10 g/L (OR = 0.82, 95% CI: 0.72–0.94, *P* = 0.0037). However, the association between sALB and the prevalence of DR was not significant when sALB was greater than 38.10 g/L (OR = 1.12, 95%CI: 0.921.35, *P* = 0.2637. Table 3).

**Table 1:** Characteristics of the Study Participants

Variable	Without DR (*n* = 259)	With DR (*n* = 167)	P-value
Age (years) *	58.08 ± 13.70	60.41 ± 12.90	0.081
Male sex, n (%) #	162 (62.55%)	78 (46.71%)	0.001
DM duration (years) ‡	6.00 (1.00–12.00)	10.00 (7.50–18.00)	< 0.001
BMI (kg/m^2^) ‡	25.1 (22.8–27.0)	24.0 (22.2–26.5)	0.016
SBP (mmHg) ‡	133.00 (124.00-147.00)	140.00 (125.50-159.50)	< 0.001
DBP (mmHg) *	80.22 ± 11.60	79.60 ± 11.49	0.588
Hypertension, n (%) #	114 (44.02%)	94 (56.29%)	0.013
Serum albumin (g/L) ‡	38.20 (36.30–40.10)	36.90 (34.40–39.70)	0.002
HbA1c (%) ‡	9.40 (7.80-11.28)	9.40 (8.00-11.20)	0.987
ALT (U/L) ‡	20.00 (15.00–27.00)	16.00 (12.00–23.00)	< 0.001
AST (U/L) ‡	19.50 (16.00–24.00)	17.00 (14.00–23.00)	0.002
Acetylcholinesterase (U/L) *	8463.67 ± 2011.18	8150.35 ± 2116.33	0.126
D-dimer (ug/L) ‡	330.00 (270.00-502.50)	410.00 (290.00-705.00)	< 0.001
TC (mmol/L) ‡	4.80 (3.90–5.60)	5.10 (4.00-6.10)	0.030
TG (mmol/L) ‡	1.51 (1.11–2.19)	1.49 (0.96–2.40)	0.929
HDL (mmol/L) ‡	0.96 (0.82–1.11)	1.02 (0.85–1.21)	0.027
LDL (mmol/L) *	3.12 ± 0.91	3.33 ± 1.06	0.026
NEFA (mmol/L) ‡	0.39 (0.26–0.53)	0.32 (0.20–0.45)	< 0.001
Lipoprotein A (mg/L) ‡	112.50 (60.00-246.00)	134.00 (82.00-262.00)	0.032
Apolipoprotein A (g/L) ‡	1.14 (1.01–1.26)	1.15 (1.01–1.31)	0.287
Apolipoprotein B (g/L) ‡	0.92 (0.74–1.09)	0.97 (0.75–1.17)	0.095
BUN (mmol/L) ‡	5.37 (4.31–6.74)	6.13 (4.90–8.64)	< 0.001
Serum creatinine (umol/L) ‡	72.00 (62.40-85.75)	75.60 (62.88–103.60)	0.032
Urinary albumin (mg/L) ‡	6.77 (3.27–18.26)	28.71 (6.10-251.66)	< 0.001
Urinary creatinine (umol/L) ‡	8.23 (5.09–13.34)	5.87 (3.92–8.31)	< 0.001
UACR (mg/g) ‡	5.83 (3.24–17.51)	41.09 (9.64-295.49)	< 0.001
eGFR (mL/min/1.73 m²) ‡	92.58 (75.89-103.46)	80.09 (48.28–98.23)	< 0.001
UACR Stage (≥ Stage 3), n (%) #	11 (4.25%)	51 (30.54%)	< 0.001
CKD Stage (≥ Stage 3), n (%) #	29 (11.20%)	52 (31.14%)	< 0.001
With DME, n (%) #	0 (0.00%)	54 (32.34%)	< 0.001

DR: diabetic retinopathy; DM: diabetes mellitus; SBP: systolic blood pressure; DBP: diastolic blood pressure; HbA1c: hemoglobin A1c; ALT: alanine aminotransferase; AST: aspartate transaminase; TC: total cholesterol; TG: triglycerides; HDL: high-density lipoprotein; LDL: low-density lipoprotein; NEFA: non-estesterified fatty acid; BUN: blood urea nitrogen; UACR: urine albumin-to-creatinine ratio; eGFR: estimated glomerular filtration rate; CKD: chronic kidney disease; DME: diabetic macular edema

*For continuous variables with normal distribution, values were presented as Mean ± SD;

‡For continuous variables with abnormal distribution, values were presented as Median (Q1-Q3);

#For categorical variables, values were presented as N (%)

**Table 2:** The linear association between sALB and the prevalence of DR in different models

Variable	Crude model	Minimally adjusted model	Fully adjusted model
sALB	0.89 (0.84, 0.95) 0.0008	0.88 (0.82, 0.95) 0.0007	0.92 (0.85, 1.00) 0.0474
sALB(Quartile)			
Q1	Reference	Reference	Reference
Q2	0.58 (0.32, 1.04) 0.0662	0.49 (0.26, 0.93) 0.0292	0.58 (0.29, 1.17) 0.1291
Q3	0.38 (0.20, 0.69) 0.0016	0.31 (0.16, 0.60) 0.0005	0.40 (0.19, 0.86) 0.0184
Q4	0.44 (0.24, 0.80) 0.0074	0.39 (0.20, 0.74) 0.0045	0.50 (0.24, 1.07) 0.0730
P for trend	0.0032	0.0022	0.0742

The models were presented as OR (95%CI), P-value

Crude model: we did not adjust other covariants

Minimally adjusted model: we adjusted age, sex, and DM duration

Fully adjusted model: we adjusted age, sex, DM duration, D-dimer, LDL, serum creatinine, HbA1c, BUN, HBP, and CKD Stage

**Table 3:** The non-linear association between sALB and the prevalence of DR in the adjusted model

The inflection point of sALB	Adjusted model	P for likelihood
< 38.10	0.82 (0.72, 0.94) 0.0037	0.030
≥ 38.10	1.12 (0.92, 1.35) 0.2637	

The model was presented as OR (95%CI), P-value

Adjusted model: we adjusted age, sex, DM duration, D-dimer, LDL, serum creatinine, HbA1c, BUN, HBP, and CKD Stage

The dashed line indicates 95% CI.

## Discussion and conclusions

Previous studies had shown that sALB may be negatively correlated with the prevalence of DR. Our findings pointed to a similar conclusion. It could be explained that sALB plays an important role in reducing oxidative stress and inflammation [12]. Furthermore, we discovered a non-linear relationship between sALB and the prevalence of DR with a saturation point close to normal levels after fully adjusting for covariates, which implied that we need to pay much attention to sALB levels in type 2 diabetic patients.

sALB had a variety of physiological functions, including regulating plasma colloid osmotic pressure and transporting substances such as hormones, fatty acids, and drugs, it could also modulate microvascular permeability and resist oxidative stress and inflammation and is associated with microvascular complications in type 2 diabetes mellitus [7, 18–20]. Sphingosine-1-phosphate transported by sALB was important in maintaining normal vascular permeability. Thus, the barrier function of the vascular endothelium would be disrupted when sALB was reduced [21]. sALB can reduce oxidative stress by lowering the production of reactive oxygen and nitrogen species, which may be related to its glutathione-dependent thiol groups with peroxidase activity [22, 23]. And nitric oxide and bilirubin carried by sALB can provide additional protection against oxidative stress [24]. Moreover, sALB was crucial in inhibiting erythrocyte membrane lipid peroxidation. And chronic hypoalbuminemia would reduce serum antioxidant activity and then result in cellular oxidative damage [25]. sALB was also an anti-inflammatory agent on vascular endothelium [26]. sALB in physiological levels can inhibit the activation of nuclear factor kappa B in a glutathione-independent manner and then vascular cell adhesion molecule-1 expression and monocyte adhesion induced by tumor necrosis factor-alpha (TNF-α) were selectively inhibited. Recent animal experiments have also found that the application of sALB could decrease the expression of pro-inflammatory cytokines (TNF-α, interleukin-1, interleukin-6 C-reactive protein, and matrix metalloproteinase-8) and promote wound healing [27]. Furthermore, sALB had direct effects on neuroprotective by promoting the proliferation of microglia [7]. Correspondingly, DR was a microvascular disease caused by oxidative stress, chronic inflammation, and neurodegenerative changes [28, 29]. Therefore, the increase in microvascular permeability, as well as the decreased function of antioxidant stress, anti-inflammatory effects, and neuroprotective function triggered by the decrease of sALB levels may account for the negative correlation between sALB and the prevalence of DR.

Although sALB can be easily measured in the clinical laboratory, its clinical significance in diseases such as DR was generally overlooked. Previous researches have found a strong link between sALB and the prevalence of DR. A cross-sectional study found that sALB was negatively correlated with DR after adjusting for other covariates [20]. And Wang et al [6]. Also discovered a significant negative relationship between sALB and the prevalence of DR after analyzing data from a large sample about type 2 diabetes mellitus (*N* = 45,462) on NHANES 2011–2020. Zhang et al [14] also demonstrated that sALB may be an independent parameter for DR in type 2 diabetes mellitus. These studies all suggest that sALB may play a crucial role in the development of DR. However, these studies drew their conclusion about the association between sALB and the prevalence of DR based on the linear analysis, lacking in considering that the relationship could be nonlinear. The relationship between sALB and the prevalence of DR was possibly non-linear in the research finished by Wang et al. And our study has confirmed the nonlinear relationship and further found a saturation effect with a saturation point (38.10 g/L) close to the normal levels of sALB by curve fitting and a two-piecewise regression model. This was the primary result of our research.

There were some limitations in our research. Firstly, the data of our study came from a cross-sectional study, the level of ALB that preceded the DR observed was uncertain. Secondly, our analysis tested association instead of causation. Therefore, further prospective studies may be required to confirm our findings. Thirdly, we could not rule out the influence of liver diseases and gastrointestinal bleeding on the association between ALB and the prevalence of DR. Although the core results did not change after adjusting for the corresponding indices of liver function, we suggest that our conclusions should be cautiously applied to the population with liver diseases or gastrointestinal bleeding.

In conclusion, based on our analysis, sALB was negatively associated with the prevalence of DR and the relationship was non-linear. The inflection point was determined to be 38.10 g/L by a two-piecewise logistic regression model. When sALB levels are less than 38.10 g/L, there is an elevated risk of DR. Raising their sALB levels to a minimum of 38.10 g/L is recommended for mitigating the risk of DR. It suggested that we should carefully monitor sALB levels and take appropriate interventions if necessary in patients with type 2 diabetes mellitus in clinical practice. Further prospective researches are needed to determine whether the change in sALB levels affected the incidence of DR.

## Data Availability

The dataset was collected by Zhuang X et al. and is now available on Dryad (via: 10.5061/dryad.6kg1sd7). The datasets generated or analyzed during the current study are available from the corresponding author upon reasonable request.

## Abbreviations

sALB: Serum albumin
DR: Diabetic retinopathy
DM: Diabetes mellitus
SBP: Systolic blood pressure
DBP: Diastolic blood pressure
HbA1c: Hemoglobin A1c
ALT: Alanine aminotransferase
AST: Aspartate transaminase
TC: Total cholesterol
TG: Triglycerides
HDL: High-density lipoprotein
LDL: Low-density lipoprotein
NEFA: Non-estesterified fatty acid
BUN: Blood urea nitrogen
UACR: Urine albumin-to-creatinine ratio
eGFR: Estimated glomerular filtration rate
CKD: Chronic kidney disease
DME: Diabetic macular edema
